# IL-1α Mediated Chorioamnionitis Induces Depletion of FoxP3+ Cells and Ileal Inflammation in the Ovine Fetal Gut

**DOI:** 10.1371/journal.pone.0018355

**Published:** 2011-03-29

**Authors:** Tim G. A. M. Wolfs, Suhas G. Kallapur, Graeme R. Polglase, J. Jane Pillow, Ilias Nitsos, John P. Newnham, Claire A. Chougnet, Elke Kroon, Julia Spierings, Coen H. M. P. Willems, Alan H. Jobe, Boris W. Kramer

**Affiliations:** 1 Department of Pediatrics, School of Oncology and Developmental Biology (GROW), Maastricht University Medical Center (MUMC), Maastricht, The Netherlands; 2 Department of Neonatology, Cincinnati Children's Hospital Medical Center, University of Cincinnati School of Medicine, Cincinnati, Ohio, United States of America; 3 School of Women's and Infants Health, The University of Western Australia, Perth, Australia; 4 Division of Molecular Immunology, Department of Pediatrics, Cincinnati Children's Hospital Medical Center, University of Cincinnati School of Medicine, Cincinnati, Ohio, United States of America; University of Giessen Lung Center, Germany

## Abstract

**Background:**

Endotoxin induced chorioamnionitis increases IL-1 and provokes an inflammatory response in the fetal ileum that interferes with intestinal maturation. In the present study, we tested in an ovine chorioamnionitis model whether IL-1 is a major cytokine driving the inflammatory response in the fetal ileum.

**Method:**

Sheep bearing singleton fetuses received a single intraamniotic injection of recombinant ovine IL-1α at 7, 3 or 1 d before caesarian delivery at 125 days gestational age (term = 150 days).

**Results:**

3 and 7 d after IL-1α administration, intestinal mRNA levels for IL-4, IL-10, IFN-γ and TNF-α were strongly elevated. Numbers of CD3+ and CD4+ T-lymphocytes and myeloidperoxidase+ cells were increased whereas FoxP3+ T-cells were detected at low frequency. This increased proinflammatory state was associated with ileal mucosal barrier loss as demonstrated by decreased levels of the intestinal fatty acid binding protein and disruption of the tight junctional protein ZO-1.

**Conclusion:**

Intraamniotic IL-1α causes an acute detrimental inflammatory response in the ileum, suggesting that induction of IL-1 is a critical element in the pathophysiological effects of endotoxin induced chorioamnionitis. A disturbed balance between T-effector and FoxP3+ cells may contribute to this process.

## Introduction

Preterm delivery is the primary cause of neonatal morbidity and mortality and its incidence worldwide is increasing [1]. The most frequent association with preterm delivery is chorioamnionitis (a histological inflammation of the fetal membranes) [2]. Prenatal inflammation, commonly associated with chorioamnionitis and prematurity, is linked with adverse outcomes of the gut including poor nutritional uptake, subsequent postnatal growth restriction, necrotizing enterocolitis and late onset sepsis [3]–[8]. However, the mechanisms responsible for the association of antenatal inflammation, preterm birth and the increased incidence of intestinal disorders remain unknown.

Recently, we used a translational model of chorioamnionitis in fetal sheep to evaluate the effects of antenatal inflammation on intrauterine gut development. We showed that exposure of the preterm gut to endotoxin disrupted maturation of the gut barrier and the innate immune defence [9]. Intestinal inflammation induced by intraamniotic LPS was preceded by increased proinflammatory cytokines with an inflammatory response in the chorioamnion and the lung [10], [11]. From the early proinflammatory cytokines that are known to be produced after LPS induced chorioamnionitis, only intraamniotic injection of IL-1 mimics the lung and systemic effects of LPS [12]–[14]. Therefore, we hypothesized that IL-1 mediated chorioamnionitis would disrupt gut development.

We administered IL-1α by intraamniotic injection prior to preterm delivery and evaluated the terminal ileum as the region of the gastrointestinal tract most vulnerable to injury and intestinal pathologies including NEC [15]. Fetal ileal inflammatory responses were evaluated with immunohistochemistry to measure myeloid peroxidase (MPO), CD3 and CD4 expressing cells and the expression of FoxP3, a transcription factor required for the development and suppressive function of regulatory T-cells. Gut wall integrity was evaluated by the distribution of the tight junctional protein Zonula Occludens-1 (ZO-1) which plays a crucial role in paracellular barrier sealing. In addition, the amount of intestinal Fatty Acid Binding Protein (I-FABP) was analyzed in the gut as a marker for intestinal mucosal damage, since this small cytosolic protein is present in mature enterocytes of small and large intestines and released if the cell membrane integrity is compromised [16], [17].

## Materials and Methods

### Animals

**Figure 1:**
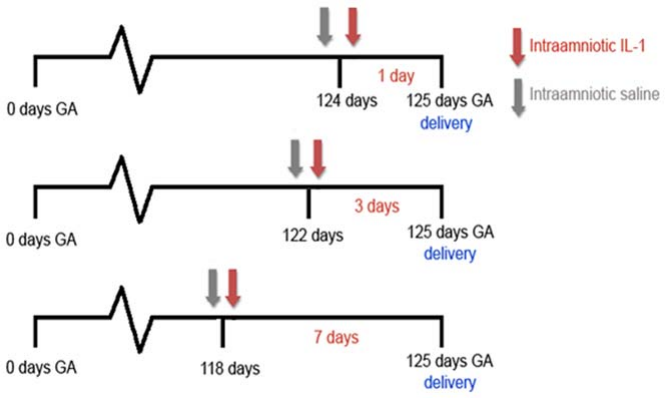
Experimental design. Antenatal inflammation was induced by a single injection of IL-1α under ultrasound guidance at 118, 122 or 124 d GA. Animals were delivered at 125 d GA and animals of the control group underwent the same procedure with an injection of saline.

The animal work for this study was performed in Western Australia and approved by the Animal Ethics Committee of the University of Western Australian and the Children's Hospital Medical Center, Cincinnati, OH (Approval ID 8D05048). Date bred Merino ewes with singleton fetus were randomly assigned to groups of six or seven animals to receive a single dose of 100 µg ovine recombinant IL-1α (Protein Express, Cincinnati, OH) at 1 d, 3 d or 7 d before caesarian delivery at 125 d gestational age (GA) (Figure 1). Control animals received intraamniotic injections with saline under ultrasonic guidance at the same timepoints (Figure 1). The 125 d GA of the fetal lambs is comparable with a human GA of approximately 27 weeks.

The biological activity of IL-1α was previously described in this model [10]. We recently described the pathological evidence of chorioamnionitis following intraamniotic IL-1 injection in this ovine model [18]. Briefly, 1–2 d following intra-amniotic injection of IL-1 alpha, inflammation in the chorioamnion was shown by histology and increased cytokine levels. In addition, increased recruitment of inflammatory cells and enhanced cytokine levels were detected in the amniotic fluid.

### Antibodies

The following antibodies were used: rabbit antibodies against human MPO and CD3 (Dakocytomation, Glostrup, Denmark), I-FABP (Hycultbiotech, Uden, the Netherlands) and Zonula Occludens protein 1 (ZO-1) (Invitrogen, San Francisco, CA); monoclonal antibodies against bovine CD4 (VMRD, Pullman, WA), human FoxP3 (eBioscience, San Diego, CA) and beta-actin (Sigma, Chicago, IL). Secondary antibodies, biotin conjugated rabbit anti-mouse or swine anti-rabbit and Texas red conjugated goat anti-rabbit were purchased from Dakocytomation and peroxidase conjugated goat anti-rabbit from Jackson (West Grove, PA). The specificity of all antibodies used in this study was extensively tested including the different secondary antibodies in combination with the appropriate isotype matched controls.

### Immunohistochemistry

Terminal ileal tissue was immersed in 10% buffered formalin for 24 h at 4°C. The formalin fixed samples were embedded in paraffin and 3 µm sections were cut. For detection of CD3 expressing cells, slides were boiled in 10 mM Na-citrate (pH 6.0) for 20 min. Endogenous peroxidase activity was blocked with 0.3% H_2_O_2_ in methanol. Slides were blocked with either normal goat serum (MPO, FoxP3) or bovine serum albumin (CD3, CD4) for 30 min at room temperature. Slides were incubated with the primary antibody of interest for 1 h at room temperature (CD3, CD4 and MPO) or overnight at 4°C (FoxP3). After washing, sections were incubated with the appropriate secondary conjugated antibody. CD3 and FoxP3 antibodies were detected with the streptavidin-biotin system (Dakocytomation) and antibodies against MPO and CD4 were detected using a peroxidase conjugated secondary antibody. Positive staining for MPO, CD3 and CD4 was visualized with 3-amino-9-ethylcarbazole (AEC, Sigma); nuclei were counterstained with haematoxylin. Immunoreactivity for FoxP3 was visualized using nickel-DAB. The numbers of cells exhibiting immunostaining were counted as follows: for MPO, CD3 and CD4, the average number of 3 different high power fields at a 200× magnification were given; for FoxP3, the sum of three high power fields (100×) was estimated. The immunohistochemical analysis was scored by 3 investigators who were blinded to the experimental conditions.

### Immunofluoresence

Immunofluoresence was performed and interpreted as described earlier [9]. Briefly, frozen ileal sections (3 µm) were incubated with anti-ZO-1 and Texas Red conjugated goat anti rabbit antibody (Jackson, West Grove, PA) respectively, followed by a 2 min incubation with 4′,6-diamino-2-phenyl indole (DAPI). The distribution of ZO-1 was recorded at a magnification of 200× using the Metasystems Image Pro System (black and white charge-couple device camera; Metasystems, Sandhausen, Germany) mounted on a Leica DM-RE fluorescence microscope (Leica, Wetzler, Germany).

### Cytokine mRNA Quantitation

Total RNA was isolated from terminal ileal tissue by Trizol/chloroform extraction. mRNA quantitation was performed using real-time PCR. Total RNA was reverse transcribed using oligo(dT) primer and Moloney murine leukemia virus reverse transcriptase (Life Technologies) according to the supplier's recommendations. cDNA was used as a template with primers and Taqman probes (Applied Biosystems, Carlsbad CA) specific to sheep sequences. The values for each cytokine were normalized to the internal 18S rRNA value. Data were expressed as fold increase over the control value.

### Western blotting

Protein sample Intestinal tissue samples were homogenized in lysis buffer (200 mM NaCl, 10 mM Tris base, 5 mM EDTA, 10% Glycerin, 1 mM PMSF, 0.1 U/mL Aprotinin and 1 µg/mL Leupeptin). Homogenates were centrifuged at 300 rpm for 10 min; supernatants were collected and centrifuged again at 10 000 rpm for 3 min. Total protein concentrations in the final supernatants were determined using the bicinchoninic acid (BCA) protein assay (Pierce Rockford, IL). To confirm equal protein loading, immunoblotting was performed with an anti-b-actin antibody. Aliquots with equal amounts of protein were heated at 100°C for 5 min in SDS sample buffer, separated on 15% SDS–polyacrylamide gels and transferred to nitrocellulose (Schleicher&Schull, Dassel, Germany). After blocking, membranes were probed with the indicated antibodies, followed by an IRDye700-conjugated secondary antibody of the appropriate species (LI-COR, Lincoln, NE, USA). Protein bands were visualized using an Odyssey Infrared Imaging System (LI-COR).

### Statistical analysis

The number of cells exhibiting immunostaining for MPO, CD3 and FoxP3 were counted per high power field. Mann-Whitney U-tests were used for between-group comparisons. Statistical calculations were made using SPSS 15.0 for Windows (SPSS, Chicago, IL) and differences were considered statistically significant at p<0.05.

## Results

### Inflammation in the fetal ileum

**Figure 2:**
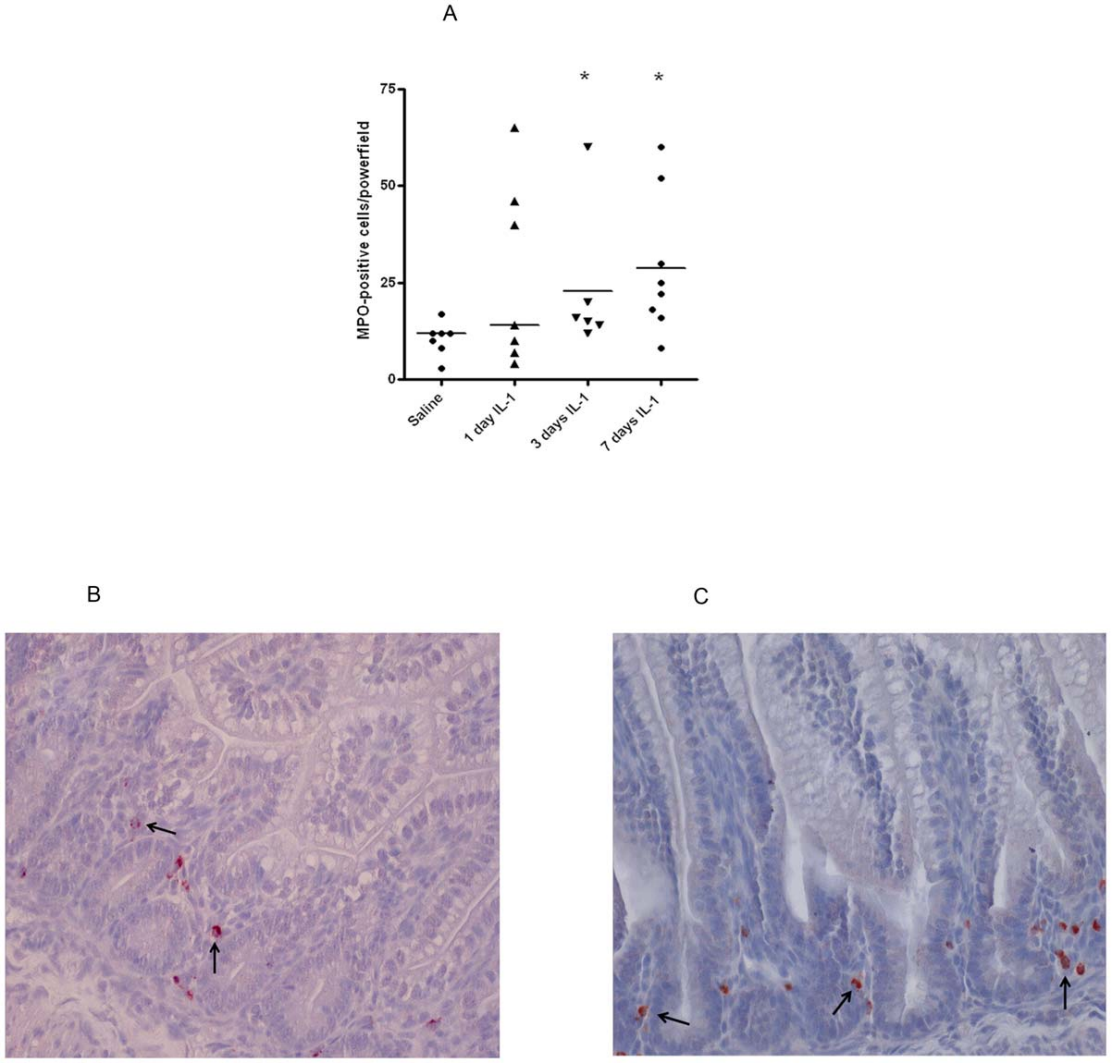
Compared to control animals, a significant (*) increase of MPO immunoreactivity in the fetal terminal ileum was seen at 3 d and 7 d after IL-1α exposure. For each experimental group, mean cell counts of MPO positive cells are given per high-power field (A). Representative sections of control animals and IL-1 treated animals for 3 d are depicted in B–C.

In preterm control animals, a small number of cells expressing MPO were detected. There was a variable increase in the number of infiltrating MPO positive cells after 1 d exposure to IL-1α (Figure 2A). However, the number of MPO+ cells was significantly increased in animals exposed to IL-1α for 3 d and 7 d when compared to control animals (Figure 2A). Representative sections of the control and 3 d groups are shown in Figure 2B–C.

**Figure 3:**
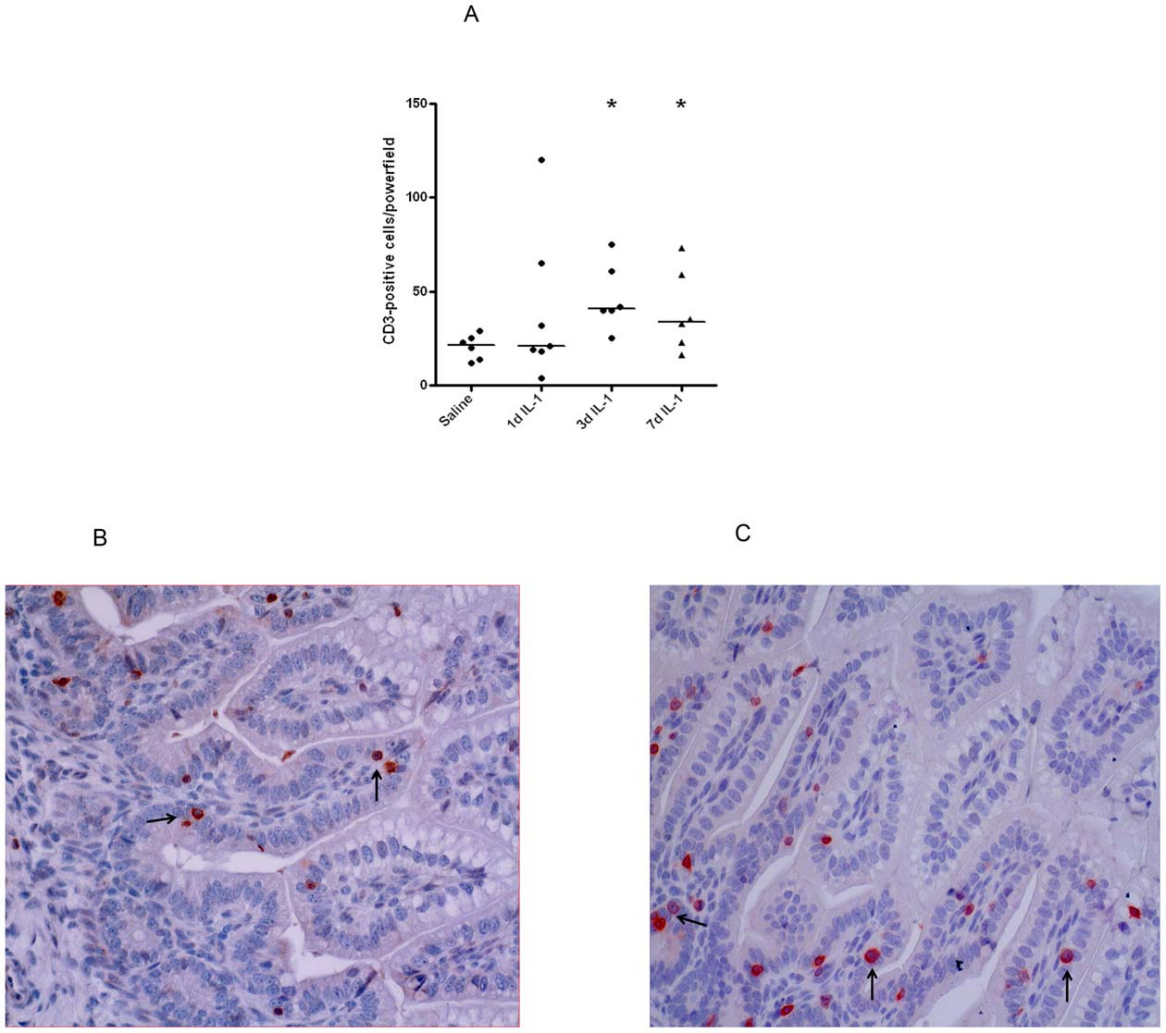
At 3 d and 7 d after intraamniotic IL-1α injection, significant increases of CD3 positive cells in the fetal terminal ileum were detected when compared with saline treated animals. For each experimental group, mean cell counts of CD3 expressing cells per high-power field are depicted (A). Representative sections of saline and 3 d IL1α treated animals are shown (B–C).

**Figure 4:**
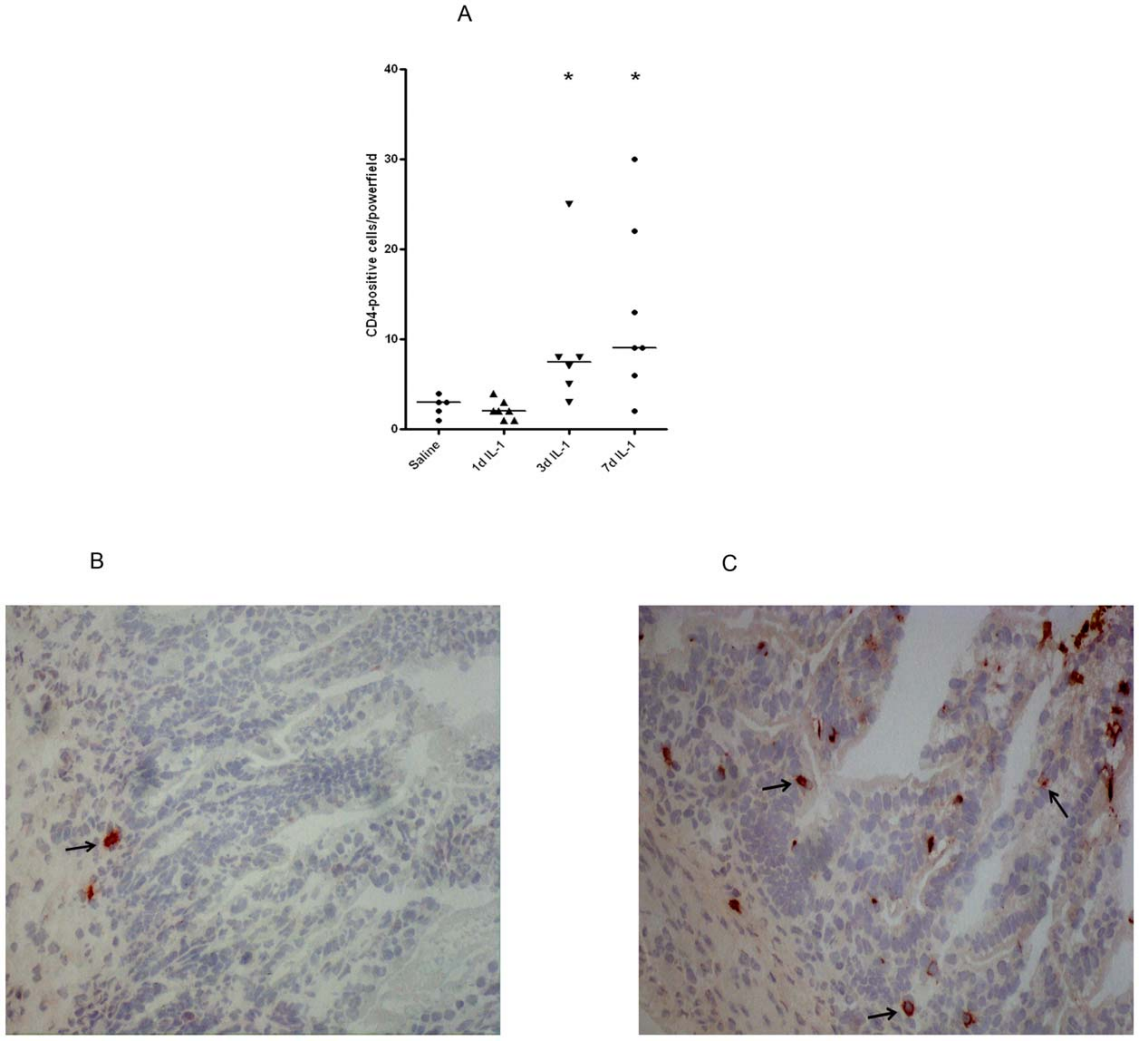
At 3 d and 7 d after intraamniotic IL-1α injection, the number of CD4 positive cells significantly increased when compared with saline treated animals. A) For each experimental group, mean cell counts of CD4 expressing cells are expressed per high-power field. B–C) Representative sections of saline and 3 d IL-1α treated animals are shown.

The influx of CD3+ and CD4+ T-cells into the fetal ileum largely paralleled the influx of MPO+ cells. In preterm lambs exposed to IL-1α for 1 d, no significant increase of CD3 and CD4 expressing T-cells in the lamina propria was observed when compared with control tissue whereas increased numbers of CD3 and CD4 expressing cells were identified at 3 d and 7 d post IL-1α treatment (Figure 3A and Figure 4A). Representative CD3 and CD4 stained ileal sections for the control and 3 d post IL-1α groups are shown in Figures 3B–C and Figure 4B–C respectively.

**Figure 5:**
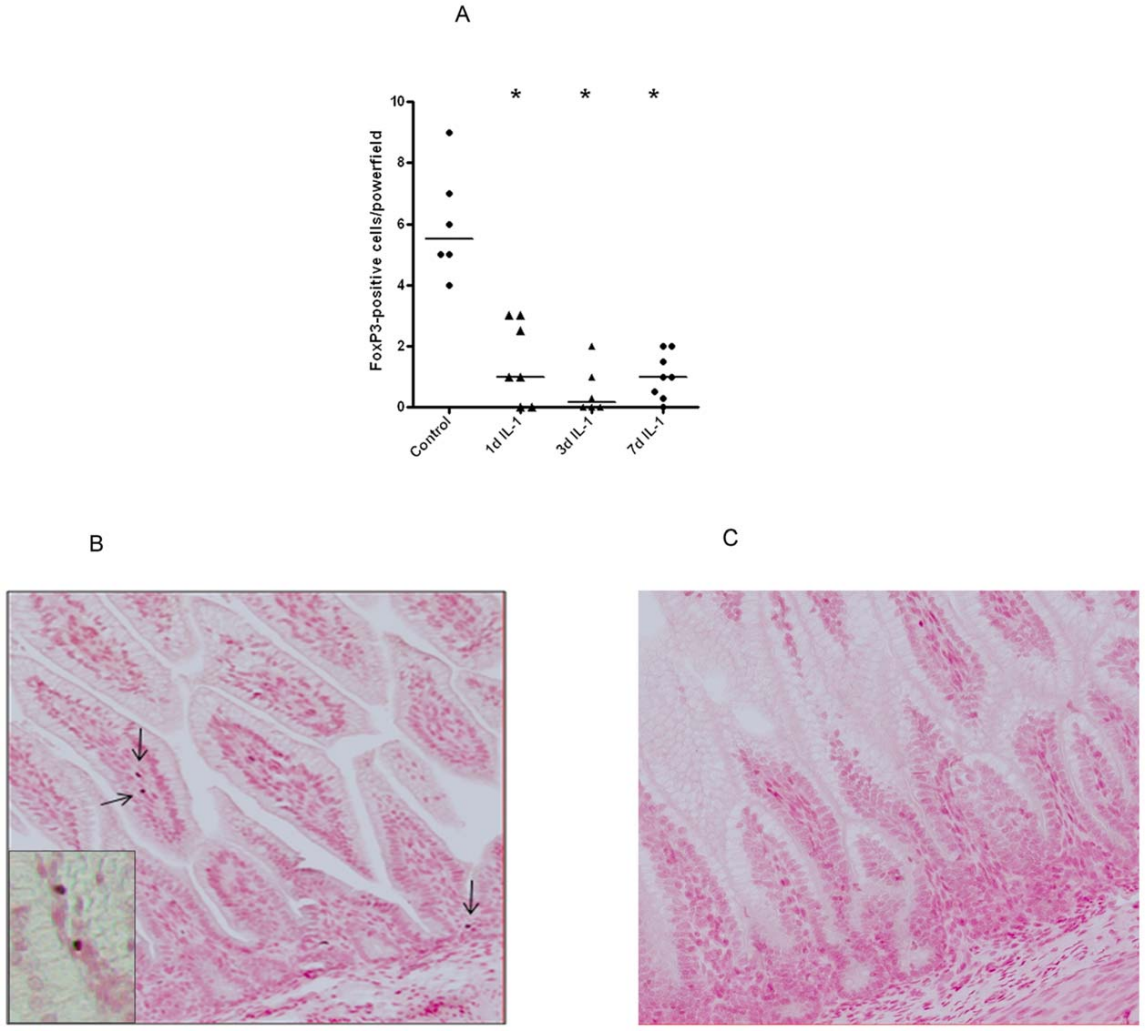
A single intraamniotic injection of IL-1α significantly reduced the number of FoxP3 expressing cells. A) Mean cell counts of FoxP3+ positive cells were measured for the sum of 3 high-power fields. Representative ileal sections of saline (B) and 3 d IL1α (C) treated animals are shown. For inset, 400× magnification was used.

Next, we identified the distribution of cells expressing the transcription factor FoxP3, the most commonly used marker for T-reg identification. When compared to control animals, FoxP3 positive cells decreased at 1 d, 3 d and 7 d after IL-1α exposure with the lowest numbers in the 3 d group (Figure 5A). Representative ileal sections for the control and 3 d post IL-1α groups are shown in Figures 5B–C.

**Figure 6:**
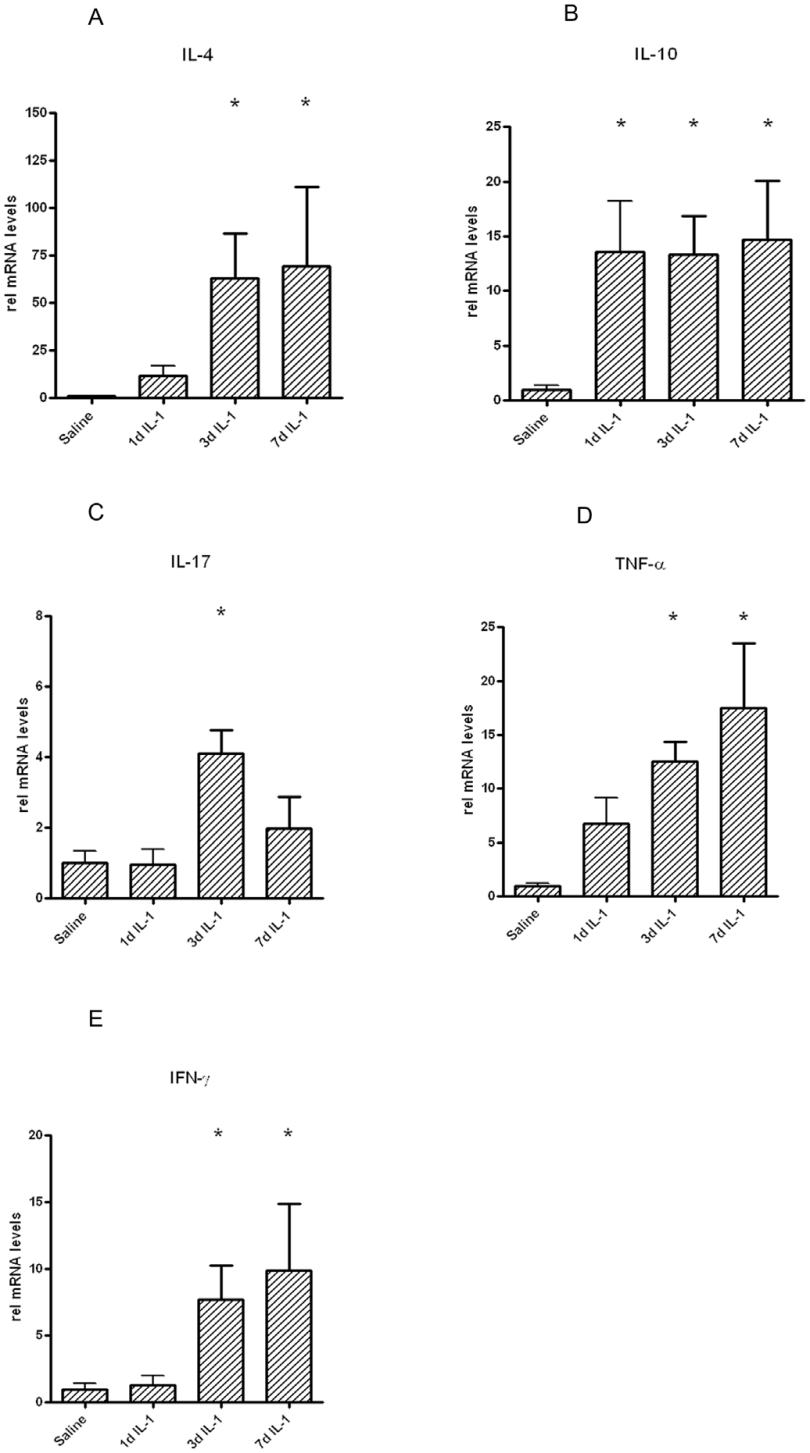
Quantification using real-time PCR assays using sheep specific primers and Taqman probes. The values for each cytokine were normalized to 18s rRNA. The mean mRNA signal in control animals was given the value of 1 and levels at each time point were expressed relative to controls (*p<0.05 vs control).

Intraamniotic injection of IL-1α strongly induced the mRNA expression of TNF-α, IFN-γ, IL-4 and IL-10 whereas IL-17 was only moderately induced 3 d post IL-1 treatment (Figure 6).

### Ileal barrier

**Figure 7:**
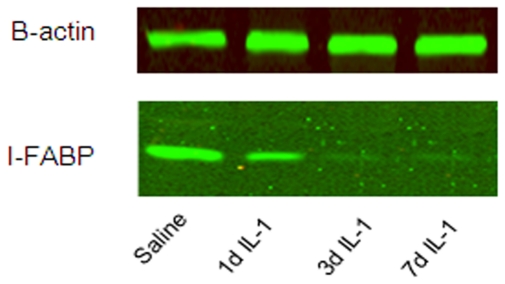
The I-FABP content in the terminal ileum is reduced within 1 d after IL-1α treatment and I-FABP levels remain low up to 7 d after intraamniotic IL-1α administration. A) Representative b-actin and I-FABP protein fragments are shown for each group. B-actin was used to confirm equal loading. Relative quantitative data were obtained by densitometric evaluation of actin and I-FABP products which were compared to a standard curve obtained by amplification of a serial dilution of a highly concentrated protein standard (*p<0.05 vs control).

Next, we asked whether the barrier integrity of the ileum was altered by IL-1α. To this end, we first analyzed intestinal levels of I-FABP, a small protein present in the cytoplasm of differentiated enterocytes. Compared to controls, intestinal I-FABP levels decreased within 1 d after IL-1α exposure reaching statistical significance at 3 and 7 d post IL-1α exposure (Figure 7).

**Figure 8:**
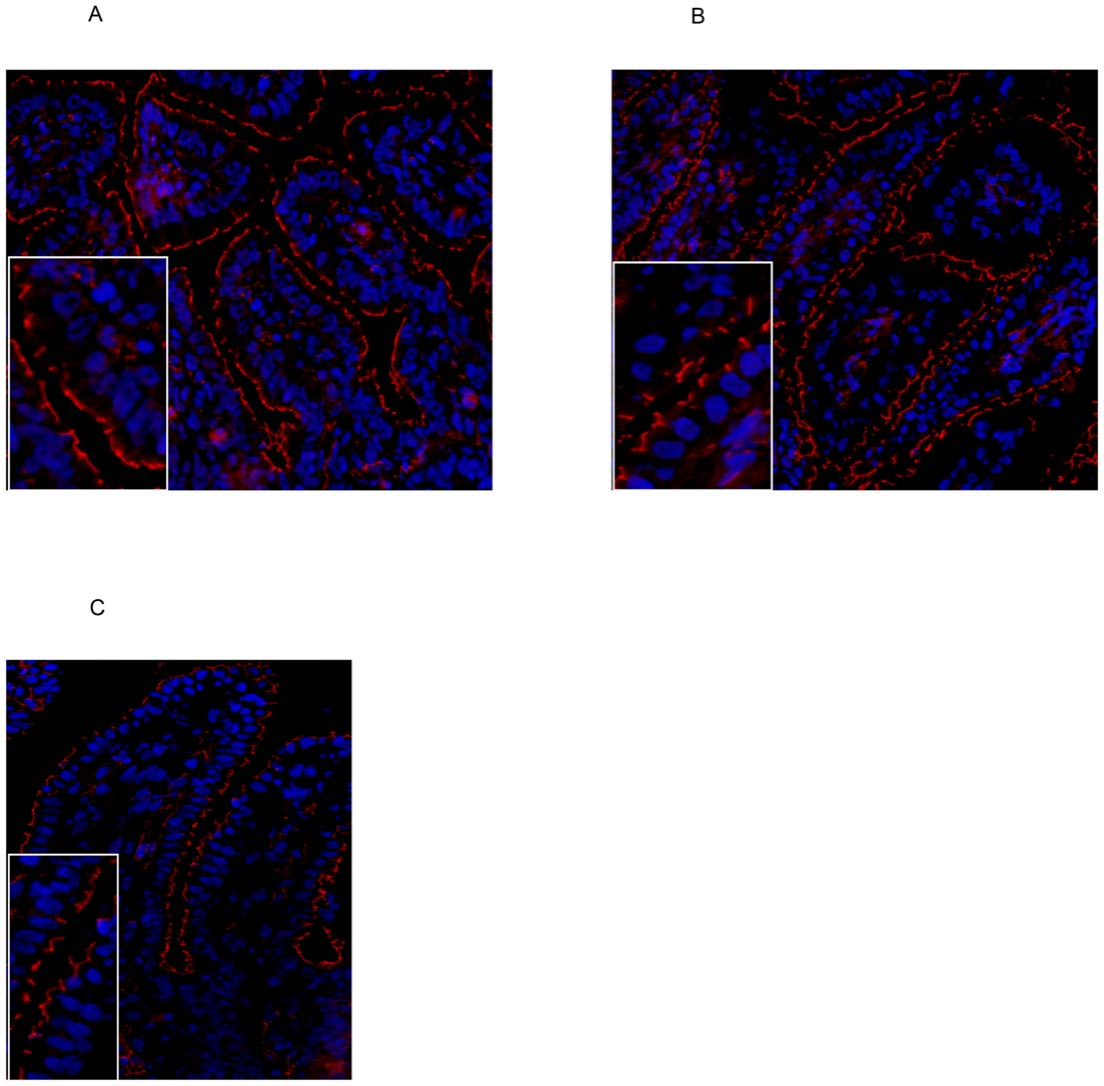
The fragmented ZO-1 distribution in preterm control animals (A) was further disrupted in lambs exposed to IL-1α for 3 d (B) or 7 d (C). Magnification 200×. For inset, 1000× magnification was used.

We also stained for the tight junctional protein ZO-1, a 225 kDa membrane bound protein which binds the transmembrane tight junction proteins occludin and claudins and links them to cytoskeletal actin [19]. ZO-1 localization was disturbed in premature control animals (Figure 8A) and this localization pattern was not changed after 1 d of IL-1α exposure (not shown). Fetuses exposed to IL-1α for 3 d (Figure 8B) and 7 d (Figure 8C) had more fragmented ZO-1 when compared with control animals.

## Discussion

We recently showed that endotoxin induced chorioamnionitis provoked an inflammatory response in the preterm ileum that interfered with maturation of the fetal intestinal immune system and ileal barrier function [9].

In the present study, we demonstrate that these harmful effects of intraamniotic endotoxin can be largely recapitulated by intraamniotic IL-1α, as indicated by the strong increase of inflammatory cytokines and concomitant barrier loss following IA IL-1α delivery. However, the influx of lymphocytes after intraamniotic LPS injection was more robust when compared to the effects of intraamniotic IL-1 injection. Therefore, this study demonstrates that induction of IL-1 is an important but not the only pathway, involved in fetal ileal inflammation and concomitant ileal barrier loss in the course of chorioamnionitis.

These results may have clinical relevance since IL-1α and IL-1β increase in the amniotic fluid during clinical chorioamnionitis and in patients with premature rupture of membranes [20]–[23]. Moreover, loss of the ileal barrier in utero such as seen in our translational model following IL-1 driven intestinal inflammation might explain the association between chorioamnionitis and the increased risk for adverse outcomes of the gut, since gut barrier loss is not only associated with poor nutrition and subsequent postnatal growth restriction, but also contributes to intestinal pathologies after preterm birth including necrotizing enterocolitis [24].

The harmful pro-inflammatory status of the ileum after intraamniotic IL-1α delivery could result from the decrease of FoxP3+ cells. This transcription factor is essential for the function of T-reg cells, which suppress T-effector cells. Although FoxP3 is the most commonly used marker for T-reg cells, its expression is upregulated in recently activated non T-reg cells in humans [25]–[27]. Therefore, we cannot rule out the possibility that FoxP3 is present in non-regulatory T-cells in our ovine model. However, since FoxP3 is decreased concomitant with the increased T-cell activation markers, this latter option seems less likely.

FoxP3+ cells decreased within 24 h after IL-1 exposure and this occurred prior to the increased influx of MPO+ cells and effector T-cells. It is tempting to speculate that loss of the inhibitory functions of T-reg cells on effector T-cells disturbs intestinal lymphoid and mucosal homeostasis, resulting in excessive immune activation and tissue damage. Alternatively, the early loss of T-reg could result in uncontrolled dendritic cell activation, which would lead to effector T-cell influx and activation. Whatever the exact target of T-reg is, such a mechanism would be consistent with earlier animal experiments and findings in humans which demonstrate that T-reg cells are critical for maintenance of intestinal tolerance to luminal antigens and for prevention of intestinal inflammation [28]–[30].

The observed depletion of FoxP3+ cells, presumed to be T-regs in fetal sheep, could be mediated by different mechanisms. These reduced numbers could be caused by increased death or decreased proliferation of regulatory T-cells [31]. On the other hand, T-reg cells could lose Foxp3 expression under inflammatory conditions. For instance, IL-4 can inhibit the generation of T-reg cells [32], [33]. Since depletion of T-reg preceded induction of IL-4 mRNA levels, one might argue that IL-4 is not responsible for this initial loss of Foxp3 expressing cells, rather it could be involved in maintenance of low T-reg numbers. Alternatively, IL-6 has been reported to induce conversion of T-reg cells to Th17 cells, thereby downregulating the expression of FoxP3 [34], [35]. However, this latter option does not seem likely since IL-17 mRNA levels are only marginally increased after intraamniotic IL-1 delivery. Another possibility is that impaired b-catenin signaling in intestinal DCs might be responsible for the observed loss of T-reg cells, since this signaling pathway was recently been shown to be required for regulation and induction of T-reg cells in the gut [36].

To our knowledge, this is the first report of (regulatory) T-cells in the preterm fetal ileum. Moreover, postnatal findings are limited. Weitkamp et al. provided data concerning postnatal developmental regulation of T-reg cells in relationship to other T cells in intestinal samples of preterm infants with and without NEC [37]. In line with our in utero findings, T-regs decreased during intestinal inflammation, but in contrast with our data, T-effector cells were decreased in the inflamed gut when compared to the healthy premature intestine [37]. This discrepancy could have multiple explanations such as the exposure of the gut to an antigen load following birth. Our model is unique as a single agonist is inducing the multiple responses in a naïve preterm fetal gut.

Interestingly, concomitant with the influx of MPO+ cells and CD3+/CD4+ effector cells, a strong induction of IL-10 was observed. Although this cytokine is critical for preservation of gut integrity as shown in IL-10 deficient mice [38], our results suggest that maintenance of FoxP3+ cells may be more critical than IL-10 induction.

Taken together, the important conclusion from the present study is that IL-1 signaling in the amniotic compartment can recapitulate pathogenesis of chorioamnionitis-induced inflammation of the ileum, and potentially its adverse outcomes. Furthermore, suggestive evidence is provided that a disturbed balance between effector T-cells and FoxP3+ cells plays a role in ileal inflammation and subsequent mucosal damage in utero following chorioamnionitis.
